# High-throughput Computer Method for 3D Neuronal Structure Reconstruction from the Image Stack of the *Drosophila* Brain and Its Applications

**DOI:** 10.1371/journal.pcbi.1002658

**Published:** 2012-09-13

**Authors:** Ping-Chang Lee, Chao-Chun Chuang, Ann-Shyn Chiang, Yu-Tai Ching

**Affiliations:** 1 Department of Computer Science, National Chiao Tung University, HsinChu, Taiwan; 2 Institute of Bioinformatics and Systems Biology, National Chiao Tung University, HsinChu, Taiwan; 3 National Center for High-Performance Computing, HsinChu, Taiwan; 4 Institute of Biotechnology, National Tsing Hua University, HsinChu, Taiwan; 5 Brain Research Center, National Tsing Hua University, HsinChu, Taiwan; George Mason University, United States of America

## Abstract

*Drosophila melanogaster* is a well-studied model organism, especially in the field of neurophysiology and neural circuits. The brain of the *Drosophila* is small but complex, and the image of a single neuron in the brain can be acquired using confocal microscopy. Analyzing the *Drosophila* brain is an ideal start to understanding the neural structure. The most fundamental task in studying the neural network of *Drosophila* is to reconstruct neuronal structures from image stacks. Although the fruit fly brain is small, it contains approximately 100 000 neurons. It is impossible to trace all the neurons manually. This study presents a high-throughput algorithm for reconstructing the neuronal structures from 3D image stacks collected by a laser scanning confocal microscope. The proposed method reconstructs the neuronal structure by applying the shortest path graph algorithm. The vertices in the graph are certain points on the 2D skeletons of the neuron in the slices. These points are close to the 3D centerlines of the neuron branches. The accuracy of the algorithm was verified using the DIADEM data set. This method has been adopted as part of the protocol of the *FlyCircuit* Database, and was successfully applied to process more than 16 000 neurons. This study also shows that further analysis based on the reconstruction results can be performed to gather more information on the neural network.

## Author Summary

It is now possible to image a single neuron in the fruit fly brain. However, manually reconstructing neuronal structures is tremendously time consuming. The proposed method avoids user interventions by first automatically identifying the end points and detecting the appropriate representative point of the soma, and then, by finding the shortest paths from the soma to the end points in an image stack. In the proposed algorithm, a tailor-made weighting function allows the resulting reconstruction to represent the neuron appropriately. Accuracy analysis and a robustness test demonstrated that the proposed method is accurate and robust to handle the noisy image data. Tract discovery is one of the most frequently mentioned potentials of reconstructed results. In addition to a method for neuronal structure reconstruction, this study presents a method for tract discovery and explores the tract-connecting olfactory neuropils using the reconstructed results. The discovered tracts are in agreement with the results of previous studies in the literature. Software for reconstructing the neuronal structures and the reconstruction results can be downloaded from the Web site http://www.flycircuit.tw. More details on acquiring the software and the reconstruction results are provided in Text S1.

## Introduction

Neurons in a fruit fly brain form numerous distinct functional circuits as in the mammalian brain. These circuits mediate the fundamental processes of vision, olfaction, locomotion, flight navigation, and complex behaviors such as feeding, learning, and memory. The neurotransmitters and molecular mechanisms that mediate these behaviors and activities are very similar to those of higher organisms and humans. It is worth studying the fruit fly brain as the initial step in understanding the functions of the neural network.

A single neuron in the *Drosophila* brain can be labeled by the Green Fluorescent Protein (GFP) [1]. Using the focus clear technique [2] or the Scale technique [3], one can employ a confocal microscope to acquire a clear image stack of the entire brain, containing a labeled single neuron. This allows one to reconstruct the structure of neurons and study the neural network of the fruit fly brain. The *Drosophila* brain contains approximately 100 000 neurons in, and therefore, reconstructing all the neuronal structures by manually tracing every single neuron is impractical. A high-throughput computer method is required.

Tracing neuron fibers is similar to tracking vasculature line structures in a 3D image volume. Previous studies on medical image processing presented related methods of tracking line structures. Bouix et al. proposed a method based on skeletonization and branch analysis [4]. Other approaches include a method based on enhancing line or edge properties and then chaining up the most likely pixels [5] and a method that attempts to find the minimal paths [6]–[8]. Compared with medical images of blood vessels, neuron images often suffer from noise and uneven resolution in the *x*, *y*, and *z* directions, and a single neuron is usually discontinuous in the image stack. Directly applying the above methods to reconstruct the neuronal structures is therefore inadequate. Researchers have recently proposed methods to trace neurons or reconstruct the neuronal structure. Al-Kofahi et al. progressively fitted and matched the primitive template structures, spheres, ellipsoids, and cylinders in the image stack [9]. However, this method did not address the situation that a neuron is fragmented in an image space. Similarly, Zhao et al. investigated the morphological characteristics of neurons [10]. Both the Al-Kofahi and Zhao's methods assumed that neuron fibers are spherical, ellipsoidal, or cylindrical; however, neuron fibers in the image stack are usually not as regular as this assumption suggests. Zhang et al. assembled many skeleton structures as a single neuronal structure [11]. However, this method considered only 2D neuron images. Lee et al. proposed a semiautomatic method for 3D neuronal structure reconstruction [12]. Peng et al. reduced the tracing problem as a variational problem by finding the geodesic shortest path [13]. Türetken et al. proposed a method based on optimizing a carefully designed energy function [14]. However, none of these methods is specific for processing numerous sets of volume data automatically.

This study presents a high-throughput computer method of reconstructing the neuronal structure of the fruit fly brain. The design philosophy of the proposed method differs from those of previous methods. We propose first to compute the 2D skeletons of a neuron in each slice of the image stack. The 3D neuronal structure is then constructed from the 2D skeletons. Biologists tend to use confocal microscopes for optimal images in a slice for human visualization; and images in two consecutive slices contain overlapped information. Consequently, a spherical object becomes oval in the image stack; that is, neurons in the image stack do not reflect the true shape of the neuron. This is the main reason we chose not to work directly on the 3D volume.

The proposed method comprises two steps. The first is the image processing step, which involves computing a set of voxels that is a superset of the 3D centerlines of the neuron. The shortest path graph algorithm then computes the centerlines. The proposed method was applied to process more than 16 000 neurons. By using a large amount of reconstructions, this study also demonstrated a result derived from the reconstructed data using the clustering technique.

The remainder of this paper is organized as follows: The Methods section details the proposed method. The Results section presents the tracing results, reconstructed neuronal structures, and an application using the reconstruction results. In this study, we used the *Olfactory Projection Fibers* from the DIADEM test data set [15] to evaluate the accuracy of the proposed method. Each image in the DIADEM test data set contains original image stacks and gold reconstructions created by experts. This study defines the distance between one reconstruction and the other, to evaluate the accuracy of the reconstruction. The accuracy analysis is also demonstrated in the Result section. The discussions are in the Discussion section.

## Results

### The Reconstructed Structure of Olfactory Projection Neuron

The neural network system of the olfactory system of *Drosophila* has received considerable attention from neural science researchers. The experiment in this study traced the axon of the olfactory projection neuron. Compared with other neurons, the axon of the olfactory projection neuron is relatively simple because it usually does not have a complex arborization structure.

**Figure 1:**
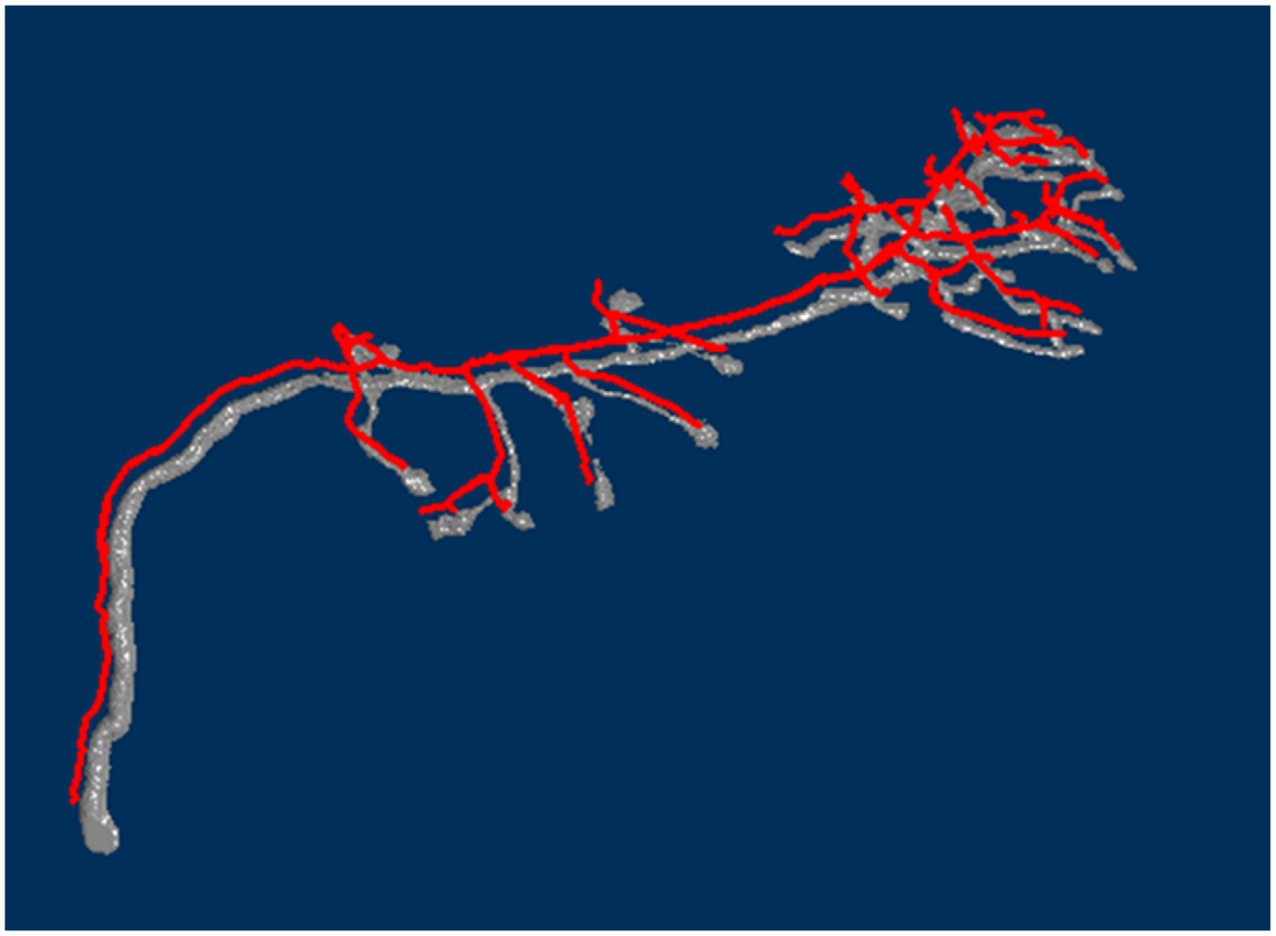
The rendered traced result (*red*) of a neuron overlaps with the volume rendering of the original image stack. For the purpose of comparison, the result is translated a little from its original position.

The datum used for the demonstration was the *Olfactory Projection Fibers* in the DIADEM dataset. For comparative purposes, the traced result and the original image were rendered in the same image, but with slight distancing (Fig. 1).

### Reconstructed Structure of Projection Neuron Connecting Optical Lobes

**Figure 2:**
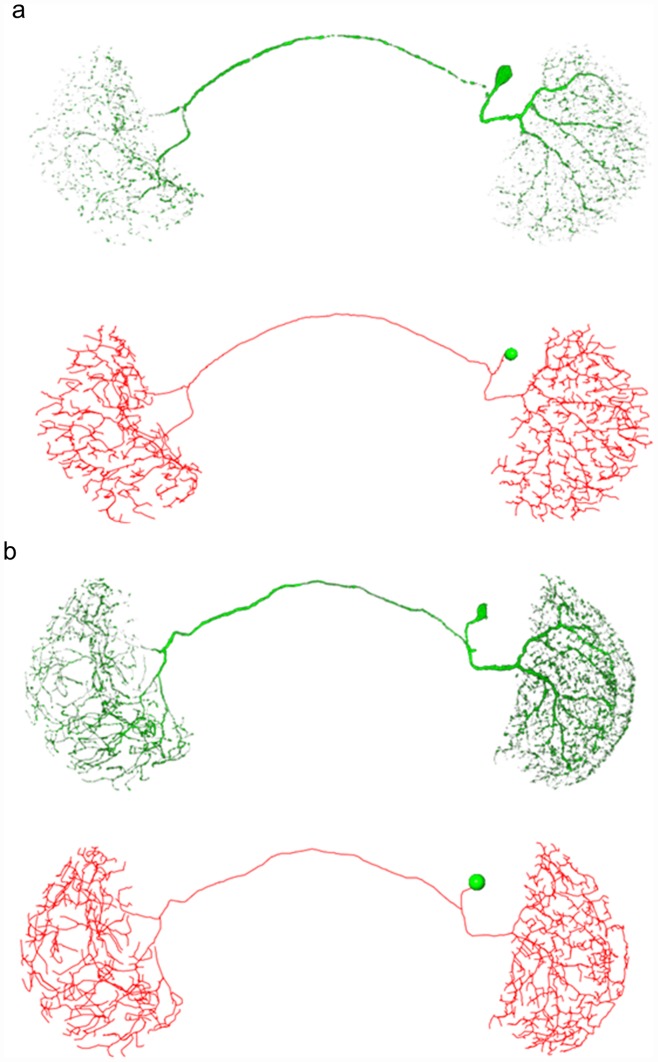
Two reconstruction results of optical nerves were demonstrated. Both (a) and (b) show the neurons (top) and the traced results (bottom). The projection neuron connecting optical lobes has dense branches and complex morphology. In addition, the intensity of the neuron in (a) has a wide dynamic range. The proposed method can manage these situations and make necessary corrections. When the whole process is completed, the reconstructions of both neurons are complete with high fidelity.

The neuronal structure is usually more complex than the olfactory projection neuron. An example of this is the projection neuron connecting two optical lobes. In this experiment, the intensity of the neuron image spreads widely, and broken branches emerge. The proposed method performs corrections necessary for producing satisfactory results. Fig. 2 shows the traced results.

### Tract Discovery

The traced results can be used to discover new information. An important application involves determining tracts in the fruit fly brain. Neuropils in the fruit fly brain are connected by neurons. The connection between two neuropils, the Antenna Lobe (AL) and the Lateral Horn (LH), were considered.

**Figure 3:**
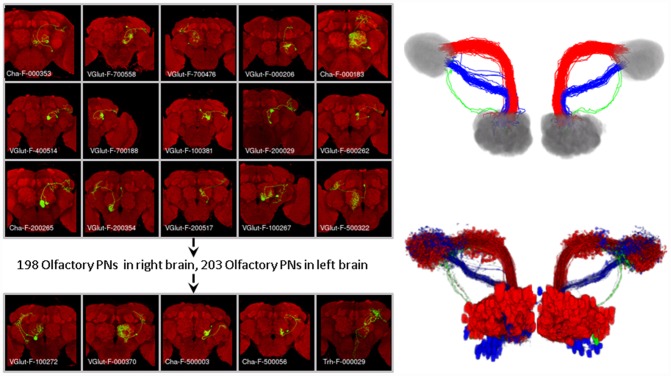
An illustration of olfactory PNs collection (left), the computed tract clusters (upper right) and the neuron image clusters overlapping the computed cluster (lower right). Totally 198 olfactory PNs in the right hemisphere and 203 olfactory PNs in the left hemisphere were selected.

Approximately 16 000 neurons were processed, and the reconstruction results were warped into a typical brain [16]. Among the reconstructed neurons, 401 traced results were selected. Of the 401 neurons, 198 neurons innervate both the LH and the AL in the right hemisphere without innervating the LH or the AL in the left hemisphere. The remaining 203 neurons innervate only both the LH and the AL in the left hemisphere. The paths connecting the AL and the LH were then extracted. Every path was evenly sampled, and hierarchical cluster analysis was applied to the sampled paths. The hierarchical cluster function supported by *R* [17] was used to complete this analysis. Text S2 provides a schematic description of the different steps in the process and a specific example of how the clusters are discovered. The results show six clusters (Fig. 3), with three on each hemisphere.

The projection neurons ascending from the antennal lobe (AL) to the lateral horn (LH) form three tracts: inner antennocerebral tract (iACT), medial antennocerebral tract (mACT), and outer antennocerebral tract (oACT) [18] [19]. These three tracts are the major message channels from the AL to the LH. The computed six clusters (Fig. 3) show that there are three clusters on both the left and right hemisphere. A comparison of these clusters to the tracts previously observed in the image data shows that they are the same as the iACT, mACT, and oACT in both hemispheres.

### Processing Time

Table 1 shows the processing time for every test datum chosen from the DIADEM test data. Except for the I/O time, it requires approximately 10 s for image preprocessing, tracing a single neuron, and the *ε*-approximation procedure. The experiments in this study were performed using a PC with an Intel Core i7 920 processor and 8 GB memory space. The actual memory usage was no more than 2 GB for all test data, including the optical nerves.

**Table 1:** Reconstruction time and accuracy.

Data	Size (voxel)	Time (sec.)	Dis(*N*_2_, *N*_1_)
OP_1	512×512×60	11.297	1.41496
OP_6	512×512×60	5.234	1.87089
OP_7	512×512×60	6.359	1.75834
OP_9	512×512×60	10.39	1.65372

### Accuracy Analysis

Taking two reconstruction results, *N*_1_ and *N*_2_, from a set of volume data containing a single neuron, we define the distance from one reconstruction to the other. The distance from *N*_1_ to *N*_2_ is defined as follows. Let *p* be a point in *N*_1_. The distance from *p* to *N*_2_ is defined as
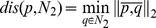
(1)In (1), 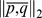 is the Euclidean distance in the image space. The distance from *N*_1_ to *N*_2_ is defined as
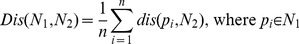
(2)This study computes the distance between the reconstruction and the ground truth to analyze the accuracy of the method. Four data sets in *olfactory projection fibers* in the DIADEM test data were used. Because the points on the neuron fibers of the ground truth are substantially denser than in the reconstruction, points on the neuron branches of the reconstruction were sampled to produce the same density of points on the neuron fibers. Let *N*_1_ and *N*_2_ be the ground truth and our reconstruction respectively. We computed *Dis*(*N*_2_, *N*_1_) for the four data sets; the distances ranged from 1.4 to 1.87. Table 1 shows a summary of the experimental results.

**Figure 4:**
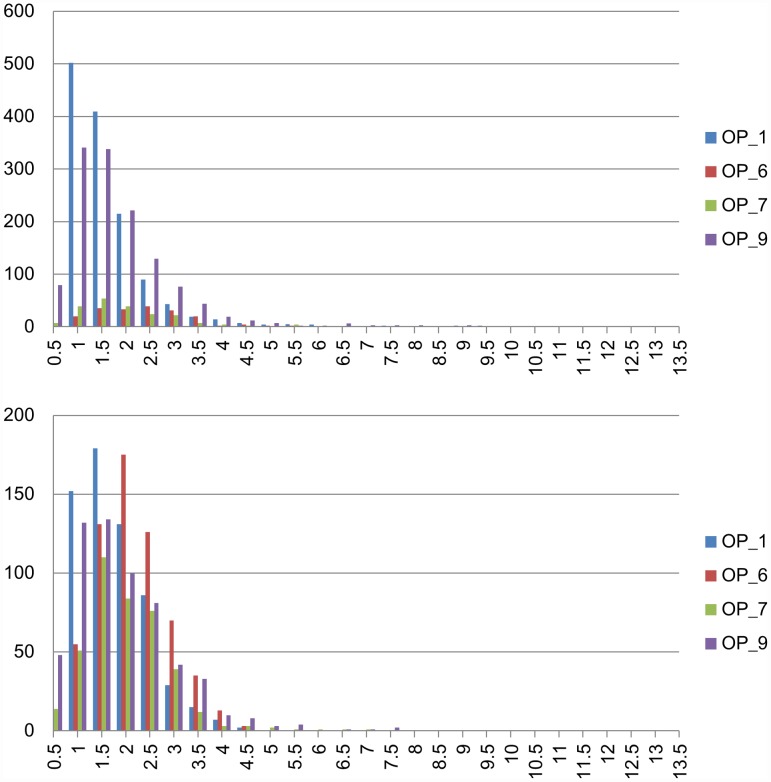
These two histograms show the distribution of the distances between points on one reconstructed result and a reference reconstruction. The references are respectively our reconstruction (top) and the ground truth (bottom).

**Figure 5:**
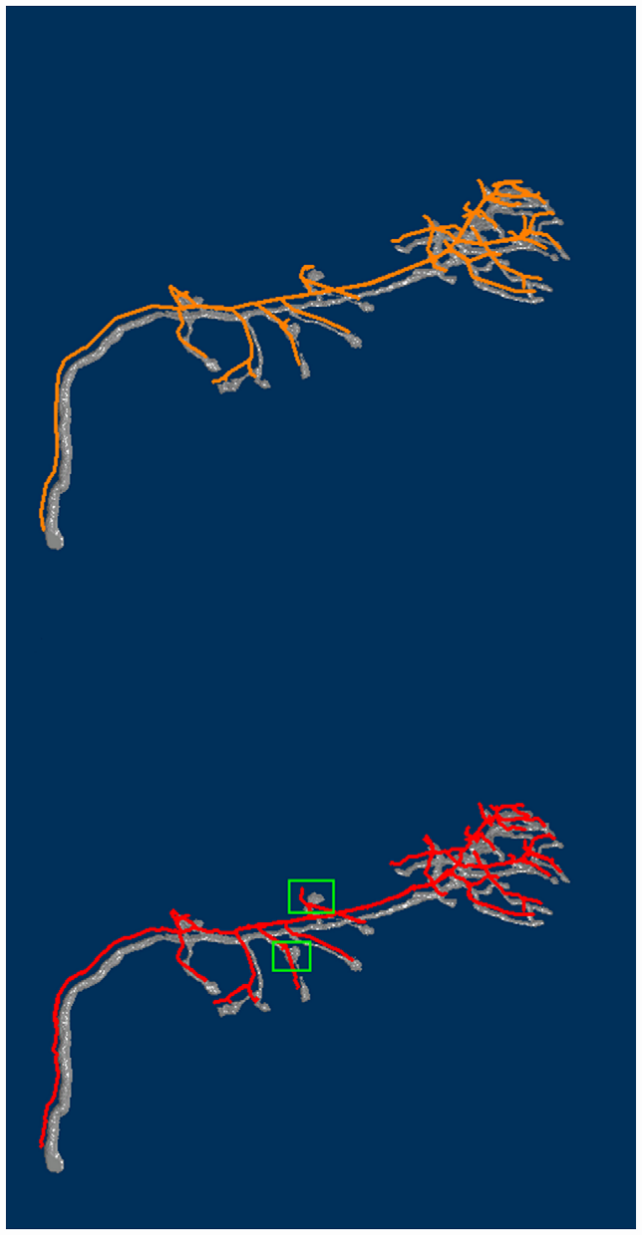
Both the gold reconstruction (top) and our tracing result (bottom) overlap with the volume rendering of the original image stack are shown. The green rectangles indicate the regions where the large distances occur.

This study also investigates the profile of 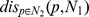 and 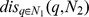. Fig. 4 shows the histograms. Most of the points are within three voxels of the reference reconstruction. The maximum difference is larger when the automatically reconstructed result is the reference. This is probably because there are large nodular structures in the projection neurons. Experts usually trace more points to the boundary of the nodular structure. However, the proposed method computes the centerline to represent the nodular structures using fewer points. Consequently, a branch in the reconstruction has a good chance of being shorter than the same branch in the ground truth. Fig. 5 shows both the gold reconstruction in the DIADEM data set and our reconstructed result overlapped with the original neuron image. Large distances appear in the green rectangular boxes. In this experiment, the distance is measured in the number of voxels. Each voxel is considered a unit cube.

### Robustness Test

**Figure 6:**
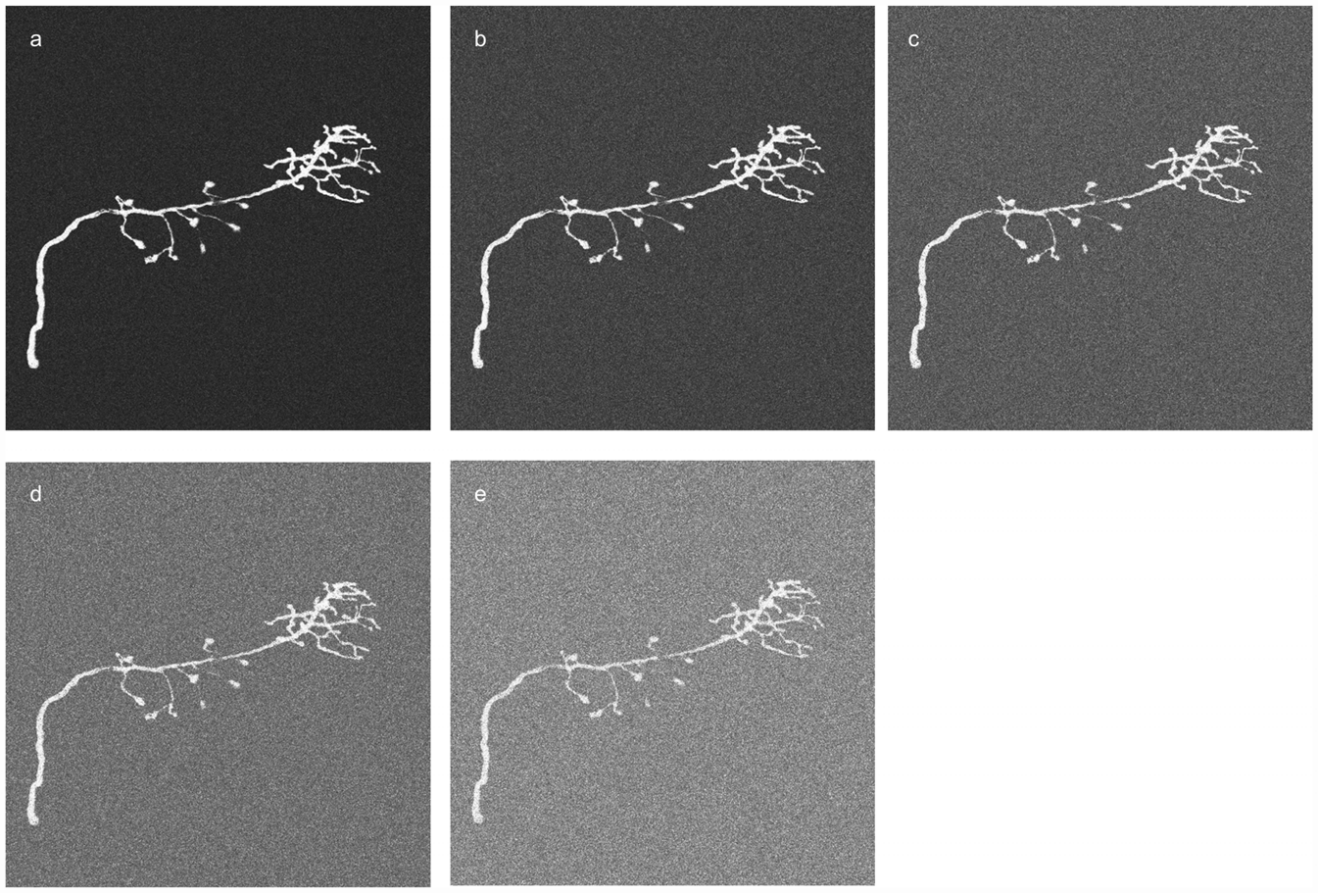
The images were contaminated by different levels of Gaussian noise: (a) σ = 20, (b) σ = 30, (c) σ = 40, (d) σ = 50, and (e) σ = 60.

The robustness test was performed by first adding Gaussian random noise to the datum, and then applying the proposed method to compute the centerlines of the neuron. We tested five cases for different noise levels. The noise levels were determined by the standard deviation of the Gaussian distribution to be *σ* = 20, *σ* = 30, *σ* = 40, *σ* = 50, and *σ* = 60. The intensity value ranged from 0 to 255. *σ* increases in conjunction with the number of visible voxels, but the percentage of visible voxels on the neuron decreases (Fig. 6), making neuron tracing more difficult. Because the image is contaminated by noise, keeping 70% of the brightest visible voxels could lead to too many noisy voxels being included. The visible voxel is defined in the Methods section. In the robustness test experiment, therefore, instead of keeping 70% of the brightest voxels, only 20% of the brightest voxels among the visible voxels were kept.

**Figure 7:**
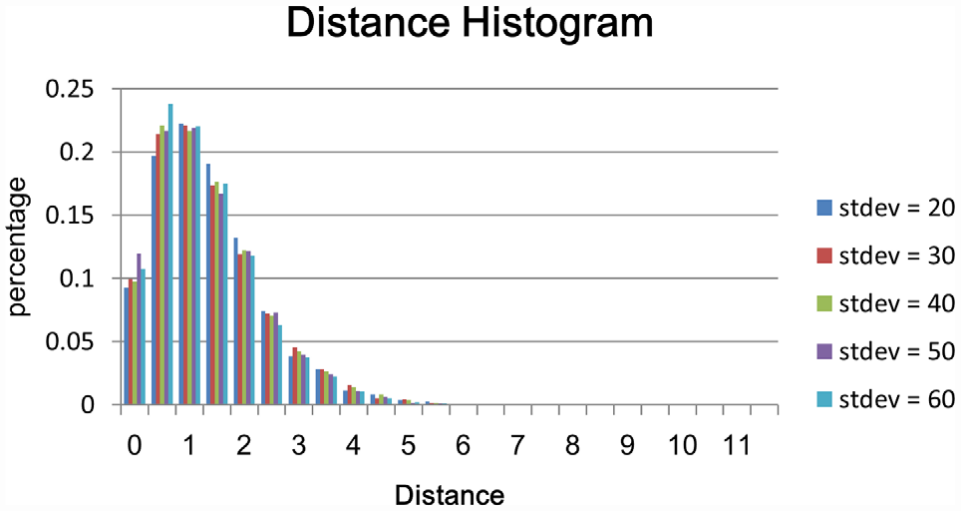
The histogram for all five different noise levels. We can find that the distributions of five noise levels are almost the same.

**Figure 8:**
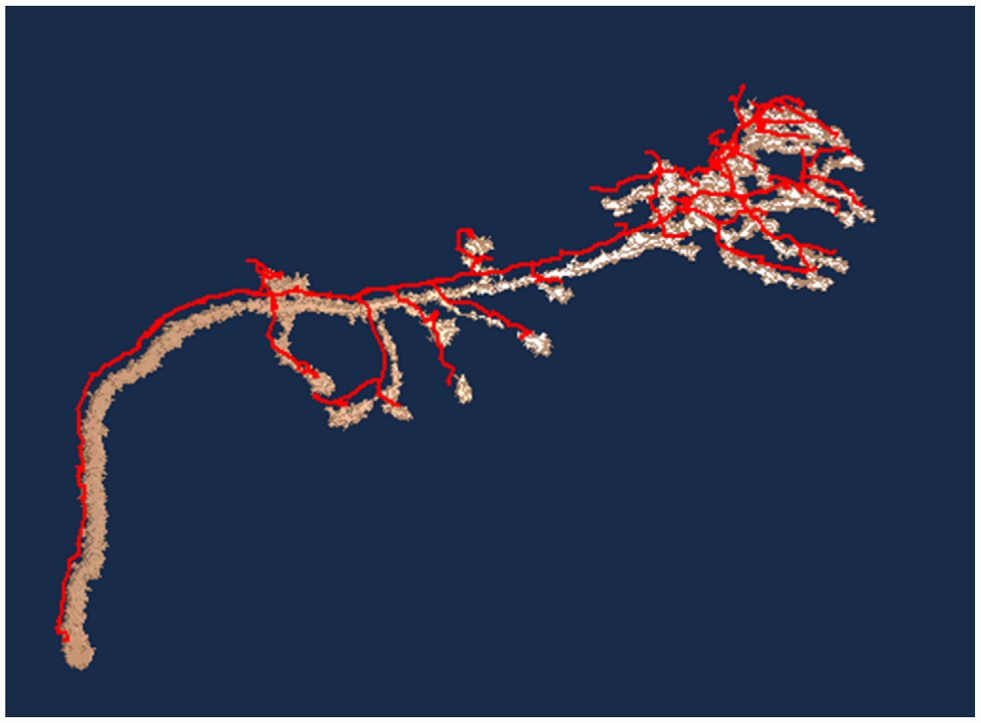
The traced result (*red*) of the contaminated, σ = 60 image stack overlaps with the volume rendering of the image stack.

To evaluate the robustness of the proposed method quantitatively in noisy images, in this study we also calculated the distance between the reconstructed result and the gold reconstruction in the DIADEM data set. As Fig. 7 shows, the distances between the reconstructions and the ground truth are still manageable, despite the noise level being at 60. Fig. 8 shows the traced results. Although the current test demonstrated that the proposed method is able to handle image data contaminated by Gaussian random noise, the algorithm will not necessarily perform well in the face of staining artifacts, which are common in biological specimens.

## Methods

### Image Acquisition

This section briefly details the technique used to label a single neuron for observing neurons using confocal microscopy [20]. A tiling heat-shock protocol in MARCM labeling was adapted to cover most neurons born at different times. Animals were kept in a 37°C water bath for 3 min to 60 min, depending on the Gal4 driver used, with 50% overlapping periods throughout the entire development from embryo to adult eclosion. In each case, GFP expression was controlled by a specific Gal4 driver with the expression being dependent on the stochastic removal of a *Gal80* repressor by heat-shock-induced expression of a flippase protein during mitotic recombination at cell birth. The Gal4 lines were driven by the promoter of an essential protein for synthesis or processing of one of the following neurotransmitters: acetylcholine (*Cha-Gal4*), dopamine (*TH-Gal4*), GABA (*Gad1-Gal4*), glutamate (*VGlut-Gal4*), octopamine (*Tdc2-Gal4*), or serotonin (*Trh-Gal4*). Thus, an individual neuron of putative birth time and neurotransmitter type was labeled.

Sample brains were imaged using a Zeiss LSM 510 confocal microscope with a 20× C-Apochromat water-immersion objective lens (N.A. value 1.2, working distance 220 µm). The following settings were used in image acquisition: scanning speed 7, resolution 1024×1024, line average four times, zoom 0.7, and optical slice 1 µm. The voxel size of *x*∶*y*∶*z* is 0∶33×0∶33×1 µm. The resolutions of the image stacks in the *Olfactory Projection Fibers* of the DIADEM data set were the same.

All the data except those provided in the DIADEM data set were acquired by neural biologists in the Brain Research Center, National Tsing Hua University, Hsinchu, Taiwan. Every image of a single neuron in 3D was segmented out of background brain tissues semi-automatically with the help of software. Human aids were provided to visually identify and select the neuron structure in the image. There are cases that multiple single neuron images were labeled from one brain. If these neurons could be clearly discriminated in 3D with ease, the neuron images were also segmented semi-automatically. Mostly, it took just few minutes to complete this step but a small number of difficult cases need more than 10 minutes.

### Image Preprocessing

**Figure 9:**
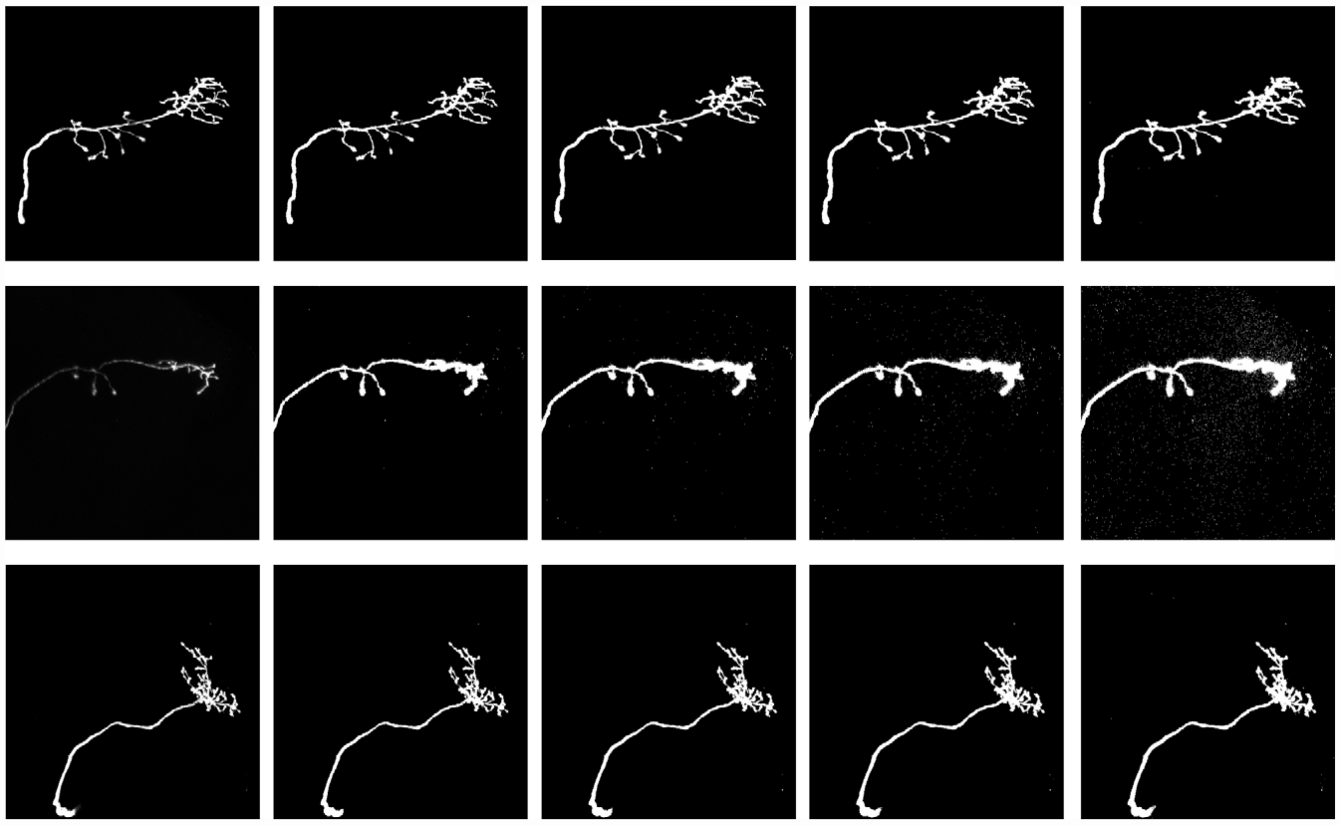
Three data of *Olfactory Projection Neuron* in the DIADEM data set are used to demonstrate the binarization method. The neurons are rendered by the maximum intensity projection (MIP) method. Every row shows a neuron undergoing different levels of binarization. From left to right, they are the original neuron image followed by the binarized results. From the second column to the last column, we kept 50%, 60%, 70%, and 80% of the brightest visible voxels where the visible voxels are voxels having a gray scale above 10. In the proposed method, we keep 70% of the brightest visible voxels, which are shown in column 4.

First, a heuristic method was used to binarize the volume data of an image stack containing a single neuron. The heuristic method was designed based on the observation that biologists tend to set up a confocal microscope for optimal human visualization of the neuron in a slice. The heuristic approach in this study segments the neuron by keeping 70% of the brightest “visible” voxels. An 8-bit grayscale voxel is “visible” if it has an intensity above 10. This method allows a sufficient number of voxels to be kept on the neuron, while maintaining a low noise level. Each row in Fig. 9 shows the images of a neuron undergoing different levels of binarization. The first column shows the original images, and the fourth column shows the results obtained by the proposed heuristic. Because the segmented result could still contain sparse noise, we remove noise by eliminating small-sized connected components in the volume. First, the 2D connected component analysis was applied to each slice. All 2D 8-neighbor connected components consisting of less than 9 pixels were removed. The 3D 26-neighbor connected component analysis was then applied to the volume to remove all 3D 26-neighbor connected components consisting of less than 30 voxels. After removing the small connected components, the 2D morphological closing operator was applied to each slice in the image stack to smooth the boundary of the neuron. This step is necessary because without it, the step that computes the 2D skeletons produces unwanted small branches for rough boundary components.

The 2D skeletons of each slice were computed before reconstructing the 3D neuronal structure. Let *B* be the volume containing the binarized neuron, and denote the set of non-zero voxels by *B_N_*. The 2D Euclidean distance transform was applied to each image slice in *B*, and the 2D skeletons for each connected component were computed based on the transformed result [21]. Let the set of points on the 2D skeletons be denoted by *Q*.

### Fragmental Line Structures Assembling

Because there is only one neuron, *Q* should form a single 3D connected component. If *Q* does not form a 3D connected component (i.e., broken branches exist), then the Minimum Spanning Tree (MST) technique is applied to compute the connected component. A weighted graph was constructed based on the 3D 26-neighbor connected components. Each vertex in this weighted graph represents a 3D connected component. There is an edge between a pair of vertices if the Euclidean distance in the image space between the closest points in two connected components is less than 5% of the largest image dimension. The edge weight is the distance between the pair of closest points. The MST of the graph was then computed using the Kruskal algorithm. Once an edge was selected during MST construction, points along the edge were sampled so that the distance between a pair of consecutive points was approximately 1. These sampled points were then added to the set *Q*. If there is more than one connected component when the process terminates, only the largest connected component is kept, and all the others are removed from *Q*. For each point in *Q*, we identify whether it is a candidate 3D end point by examining nine digital planes in the 26-neighborhood [22]. The set of candidate 3D end points is denoted by *V_E_*. The next step reconstructs the 3D neuronal structure from *Q*.

### Reconstruction of the Neuron Branches

Another weighted graph was constructed from the point set *Q*. The shortest path algorithm employed in this study is the single-source shortest path algorithm. Because a source point in the graph should be given, this study defines the center of the soma as the source. In the image, the soma is a set of high-intensity voxels forming a spherical object. Geometrically, the Chebyshev center of a set is the point within the set that is the farthest from the boundary ([23] Ch. 8). An approximated Chebyshev center for the center of the soma served as the source vertex in the graph.

**Figure 10:**
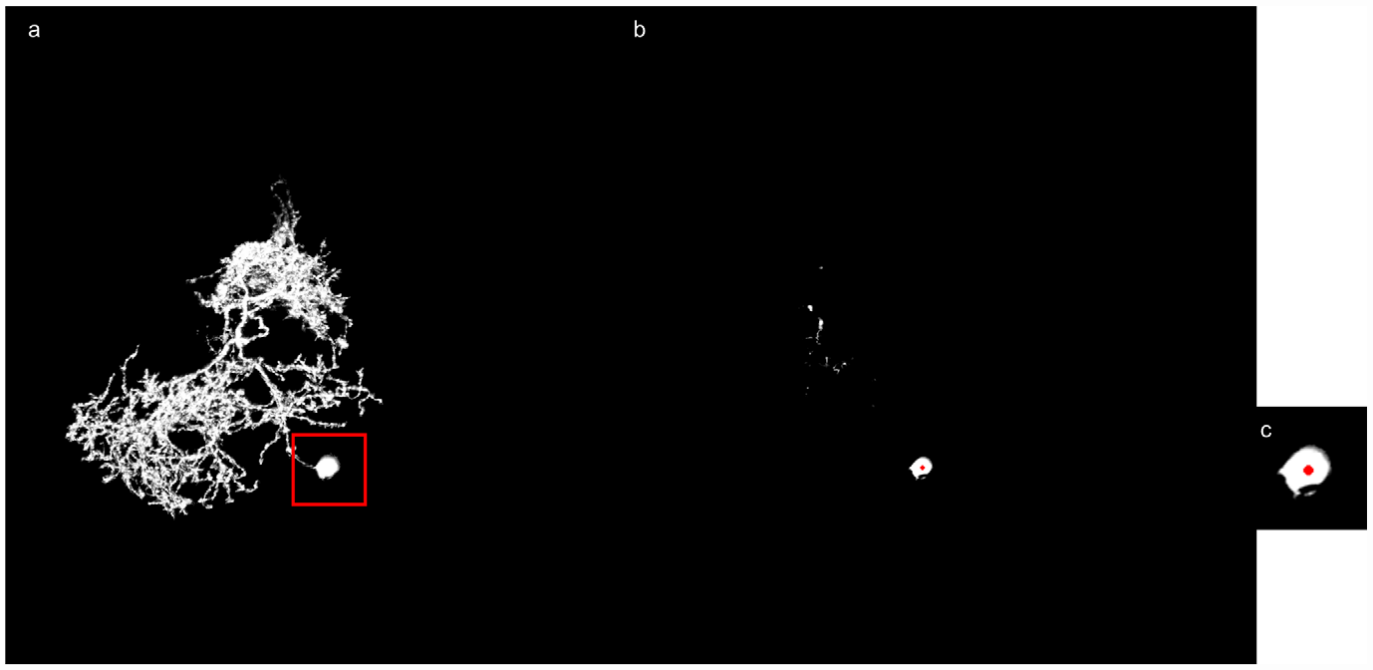
An example of the soma detection. (a) The MIP image of the original image stack. (b) Red dot indicates the center of the soma calculated by the soma detection procedure. (c) A close view of the detected soma position.

A good approximation for the Chebyshev center of the soma is a point in *Q* that is farthest from the boundary. The approximate Chebyshev center is computed iteratively as follows. For each point *p* in *Q*, we iteratively increase the radius, *r_p_*, of the sphere centered at *p* until the sphere cannot enclose points totally in *B_N_*. The center of the soma is the point *c* in *Q* that admits the largest sphere enclosing points totally in *B_N_* and the largest sum of the intensity. Fig. 10 shows an example of soma detection.

**Figure 11:**
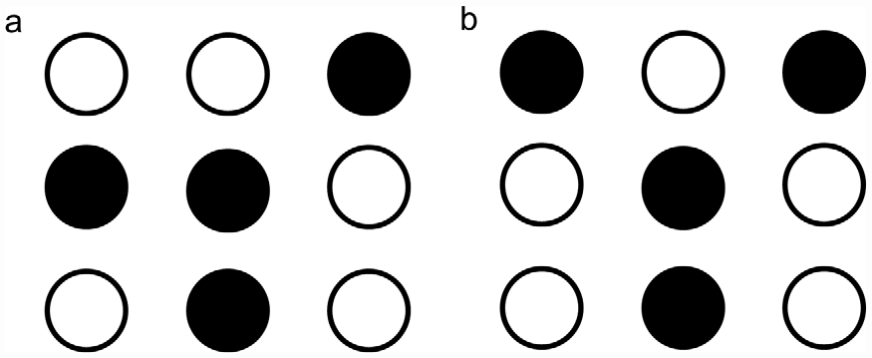
The black nodes represent the points in Q.

To push the 3D centerline toward the true center of the neuron, it is important to identify the *candidate branch points*. A 2D skeleton point is a candidate branch point if it has four or five neighboring points in *Q* and the arrangement of four is isomorphic to one of the patterns shown in Fig. 11. The set of candidate branch points is denoted by *Bps2D*.

A weighted graph *G* = (*V,E*) was constructed from *Q*. *V* is a set of vertices where each vertex is a point in *Q*. *E* is a set of edges (*p, q*), where *p* and *q* are in *Q* and neighboring to each other in the volume. The cost associated with the edge (*p, q*) is
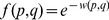
(3)where *w*(*p, q*) is as shown in (4).
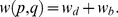
(4)In (4), *w_d_* is the Euclidean distance between *p* and *q* in the image space. The traced branches should intersect at a single branch point where a visual bifurcation point is present. To meet this requirement, an edge close to a candidate branch point has a large value for *w_b_*. Let *b_p_* be a candidate branch point. Consider the sphere centered at *b_p_* with a radius *R* = 1.0 µm. If (*p, q*) is enclosed in the sphere, then *w_b_* of (*p, q*) is *η−d*, where 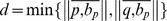. Otherwise, *w_b_* = 0. *R* is set to be 1.0 µm. This is because a sphere of the radius of 1.0 µm is usually totally enclosed in the neuron in the bifurcation region. Because the resolutions in the *x*-,*y*-, and *z*-directions are 0.33 µm, 0.33 µm, and 1.0 µm, respectively, a 6×6×2 box was used to approximate the sphere. To guarantee that *w_b_* is positive, set *η* = 10.

In (4), the edges close to a branching point have a large *w_b_* = *η−d*. Thus, these edges have a heavy weight, *w*(*p, q*), and light cost, *f*(*p, q*). When the shortest path algorithm is applied, these edges tend to become a part of the shortest path; thus, the constructed shortest paths tend to keep the appropriate branch points of the neuron branches.

**Figure 12:**
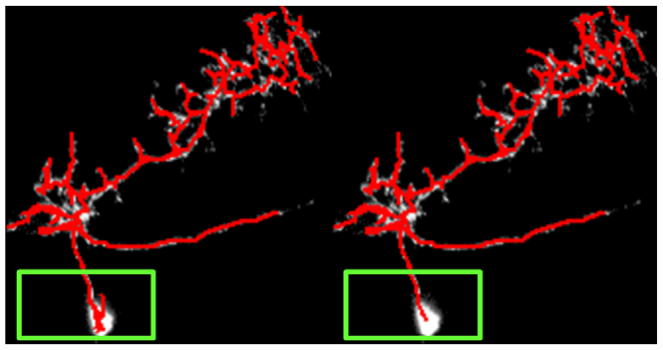
The results of before (left) and after (right) removing short branches inside the soma are demonstrated. The green rectangles indicate the somas.

Given the weighted graph and the source vertex *c*, the shortest paths from *c* to all the points in *V_E_* can be computed by applying the Dijkstra algorithm. Each path from *c* to a vertex in *V_E_* is called a branch. A branch should be removed if the ratio between its length and the length of the longest branch is less than 0.2. Such short branches are usually branches in the interior of the soma. These branches in the soma are not neuron fibers and they should be removed. Fig. 12 shows the results of before and after removing short branches.

The neuronal structure is reconstructed by iteratively selecting a branch obtained in the previous step, followed by post-processing as described in the following steps. Iterations stop when *V_E_* is empty. A list (*BpList*) that stores the branch points formed during the reconstruction process is required.

1. Select the longest branch, *P*, which is a branch from *c* to a point *p* in *V_E_*. Remove *p* from *V_E_*. If *BpList* is not empty, compute the physical distances between the points on *P* and the points in *BpList*. Along the path from *p* to *c*, let *b* be the first point, such that 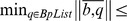
1.0 µm. In this case, *b* is considered the same as *q*. Furthermore, *P* can be divided into two paths: from *c* to *b*, and from *b* to *p*. The path from *c* to *q* is a subpath of a previously reconstructed path, *P′*. In this case, the branch *P* is modified to be the path from *c* to *q* of *P′*, and *b* in the path from *b* to *p* is replaced by *q*. If no such pair exists, look for another pair (*b′, q′*), in which *q′* is from *Bps2D* and *b′* is from the path along *p* to *c*. Let *b′* be the first point, such that 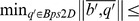
0.75 µm, and replace *b′* by *q′* on the branch.
2. If *P* shares a common subpath from *c* to *bp* with a previously reconstructed branch and *bp* is not in *BpList*, then *bp* is inserted into *BpList*.
3. Because the skeletons were computed in 2D slices, false candidate 3D end points exist. In this study, the candidate 3D end points close to *P* are considered to be false end points, and the branch is removed if the distance between its end points and *P* is less than 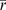, where 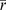 is the average of 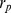, 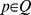.

### Polygonal Path Approximation

**Figure 13:**
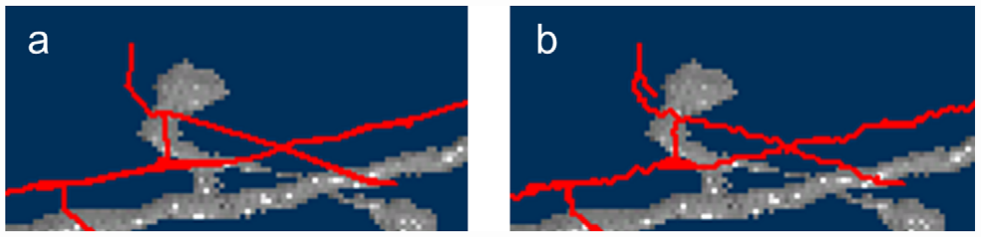
A zoom-in view of the traced result. Red lines show the tracing results. The lines in (a) are zigzag shaped. However, they become smooth when the ε-approximation method is applied (b). The *ε* was 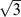.

The 3D centerline obtained by applying the shortest path algorithm was not smooth because the graph is a grid graph (Fig. 13(a)). To construct smooth centerlines, an *ε*-approximated polygonal path was calculated to approximate the centerline computed by the shortest path algorithm. An *ε*-approximation of a polygonal path has fewer points on the path, within a small deviation, *ε*, from the original polygonal path (Fig. 13(b)). Text S3 details the algorithm for computing the *ε*-approximation. All experiments in this study adopted a value of 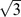 for *ε*.

For the convenience of reproducing our results, the parameters used in all these steps are summarized in Table 2.

**Table 2:** Parameters for reconstruction.

	Image Preprocess	Fragmental line structures assembling	Reconstruct the 3D centerlines	Polygonal path approximation
Initial threshold level	10, in a 256-gray scale image	_	_	_
Threshold for the 2D 8-neighbor CC	9	_	_	_
Threshold for the 3D 26-neighbor CC	30	_	_	_
Maximum edge weight to connect two components	_	5% of the largest image dimension	_	_
Radius *R*	_	_	1.0 µm	_
*η*	_	_	10	_
Ratio for determine branches in the soma	_	_	0.2	_
Threshold for connecting to a point in BpList	_	_	1.0 µm	_
Threshold for connecting to a point in Bps2D	_	_	0.75 µm	_
ε for path approximation				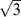

## Discussion

In this paper, we presented a computer method for reconstructing neuronal structure from an image stack. Based on the fact that biologists tend to use confocal microscopes for optimal images in a slice for human visualization, we proposed processing 2D slice images first. 3D neuronal structures were then constructed from the processed 2D images. Using this strategy, a high-throughput method was designed. More than 16 000 neurons were reconstructed and stored in the database [16]. A few of the reconstructed neurons were incorrect, mainly because the resolution of the optical microscope is not sufficient to distinguish dense branches.

The features used to design the weights of the edges (Eq. 4) were extracted from the 2D skeletons. One of the features is the branch point; however, the proposed template matching method is unable to detect all branch points. When a slice passes through the branch point, and the two branches are respectively above and below the slice, we are not able to detect the branch point. This problem may cause errors in the location of the branch point.

Another weakness of the proposed method is related to detecting delicate structures. The candidate 3D end points were obtained from the 2D end points, and the end points close to a branch were removed. Some small branches could therefore be considered as noise and ignored. Thus, the reconstruction by the proposed methods is inadequate for a study for which the details of a neuron are extremely important, e.g. the study of neuron dynamics [24].

Currently, the DIADEM [15] data set is widely used in the study of neuron reconstruction for accuracy evaluation. A scoring system is provided. We used the DIADEM data set as ground truth to evaluate the accuracy of the proposed method; however, reconstructions obtained by the proposed method did not achieve a good DIADEM score. The reasons are:

1. The neuron branches obtained were based on the 2D skeletons in each slice. Thus, neuron branches obtained by the proposed method are shorter than those traced by experts.
2. Our approach tends to merge branch points in a small region to a single point. The proposed method could ignore some branch points in the ground truth. However, each branch point is highly weighted in the DIADEM metric so that our reconstructed neuron could receive a serious penalty.
3. The DIADEM ground truth constructed by experts was obtained using NeuroLucida. The traced neurons were smoothed by spline interpolation. Coordinates of points on the neuron branches are real numbers. We used a polygonal line to approximate a neuron branch; coordinates of the points are integer numbers. Consequently, a large error could occur in estimating the radius of the neuron branches of our reconstruction.

Although neurons constructed by the proposed method cannot achieve a good DIADEM score, nevertheless, as shown in the Results section, the reconstructed results are suitable for further studies. In conclusion, the reconstructed neurons are sufficiently reliable to support the analysis of the neural network.

## Supporting Information

Text S1 An introduction to the reconstructed data.
(DOCX)

Text S2 Steps for constructing neural tracts.
(DOCX)

Text S3 Details of polygonal path approximation method.
(DOCX)
