# Supplementary material for: High-throughput Computer Method for 3D Neuronal Structure Reconstruction from the Image Stack of the Drosophila Brain and Its Applications
Source: PLoS Comput Biol. 2012 Sep 13;8(9):e1002658. doi: 10.1371/journal.pcbi.1002658 (PMC3441491; doi:10.1371/journal.pcbi.1002658)
Supplement: Text S1 — An introduction to the reconstructed data. (DOCX) [file pcbi.1002658.s001.docx]

**S.1 Introduction to reconstructed data**

All the image data (.LSM) and the reconstructed neuronal structures (.am) can be downloaded from the Web site: <http://www.flycircuit.tw> (Fig. S1). To access these files, the user has first to apply for an account. Before tracing a single neuron, the soma position in the image space must be defined. As well as a program to detect the soma position, we also provide the information about soma positions indicated by experts. This information can be found at the Web site: http://www.flycircuit.tw/flycircuit.soma. To convert a reconstructed result to Amira lineset file format, the bounding box information is necessary. The user can find the bounding box information at the Web site: <http://www.flycircuit.tw/flycircuit.boundingbox>

The physical voxel size in the image space is 0.33 μm x 0.33 μm x 0.33 μm. The physical voxel size of each reconstructed datum can be calculated from the bounding box information and the image dimensions. Another issue concerning the reconstructed result is that it is not consistent from machine to machine. At least, according to our experiment, a different version of STL and different computer architecture could lead to minor differences.

Currently two plate forms, Windows and Linux are supported and three versions of software are on-line:

1. Windows 32 Bit:

<http://www.flycircuit/NT/Win32.zip>

1. Windows 64 Bit:

<http://www.flycircuit/NT/Win64.zip>

1. Linux 32 Bit:

http://www.flycircuit/NT/Linux_x86.zip

The instructions for each version of the programs are included in the zip file.


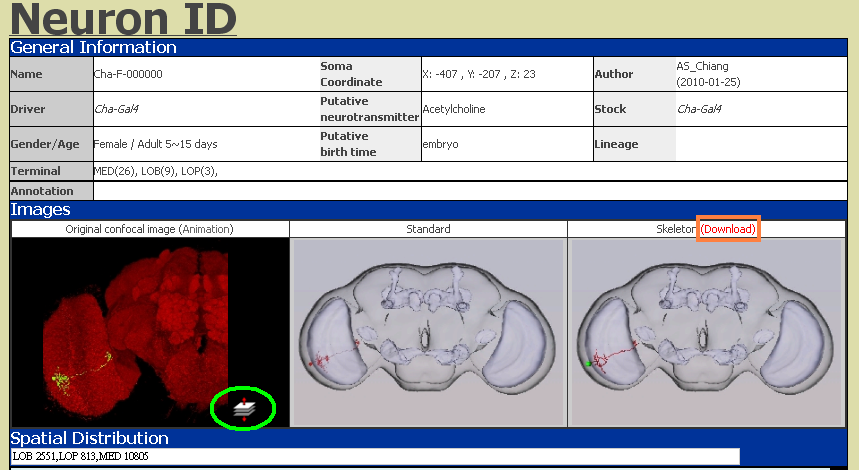


Figure S1: The reconstructed data can be downloaded via the link “Download” (*orange rectangle*) and the original image data can be downloaded via the iconic link (*green circle*).
