# Supplementary material for: High-throughput Computer Method for 3D Neuronal Structure Reconstruction from the Image Stack of the Drosophila Brain and Its Applications
Source: PLoS Comput Biol. 2012 Sep 13;8(9):e1002658. doi: 10.1371/journal.pcbi.1002658 (PMC3441491; doi:10.1371/journal.pcbi.1002658)
Supplement: Text S2 — Steps for constructing neural tracts. (DOCX) [file pcbi.1002658.s002.docx]

**S.2 Steps for constructing neural tracts**

Here, we take four neurons connecting ipsilateral medulla and contralateral lobula plate (Fig. S3(a1)-(a4)) as a simple example to demonstrate how to construct the neural tracts.

1. In each of the two neuropils, all terminals (brown spheres) and their average positions (blue spheres) were determined (Fig. S4(b1)). The shortest path connecting the two selected terminals that are closest to the two average terminal positions was found (Fig. S4(b1)).
2. For simplicity, the cell body and internal paths in each of the two neuropils were removed (Fig. S4(b2)-(b3))
3. Each of the 4 tracts was cut into 99 fragments of equal length. We then calculated the average distance of 100 points between tract pairs, and created a distance matrix table (Fig. S4(b4)).
4. We used the function (Hclust) in *R* to calculate the matrix table. Finally, the tracts of neuron VGlut-F-300388 and VGlut-F-800007 were bundled into a single tract; the tracts of neuronVGlut-F-000192 and VGlut-F-000429 were bundled into another single tract.


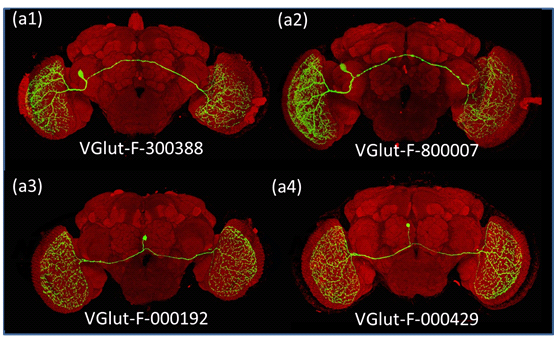


Figure S3: Four neuron tracts are used for the demonstration


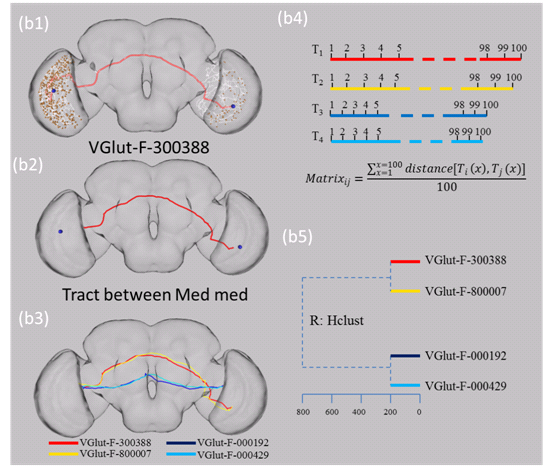


Figure S4: Steps for constructing neural tracts
