# Supplementary material for: High-throughput Computer Method for 3D Neuronal Structure Reconstruction from the Image Stack of the Drosophila Brain and Its Applications
Source: PLoS Comput Biol. 2012 Sep 13;8(9):e1002658. doi: 10.1371/journal.pcbi.1002658 (PMC3441491; doi:10.1371/journal.pcbi.1002658)
Supplement: Text S3 — Details of polygonal path approximation method. (DOCX) [file pcbi.1002658.s003.docx]

**S.3 Polygonal Path Approximation**


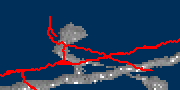

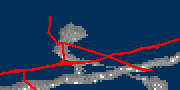


1. (b)

Figure S2: A zoom-in view of the traced result. (a) Red lines show the tracing result; (b) The *ϵ*-approximation of the tracing result, where *ϵ* =$\sqrt{3}$.

Given a polygonal path *S* =*< v*_0_*, . . . , v_m_ >* and an error bound *ϵ*, we look for a polygonal path, $\tilde{S}$, that is an *ϵ*-approximation of *S*. $\tilde{S}$ =*< u*_0_*, . . . , u_m_ >* optimally *ϵ*-approximates *S* if $\tilde{S}$ meets the following criteria.

1. Vertex set of $\tilde{S}$ is a subset of *S*.
2. Let *u_i_* = *v_j_* and *u_i_*_+1_ = *v_k_, i* = 1*, . . . ,m -*1, the distance between any vertex on the polygonal path *< v_j_ . . . , v_k_ >* and the line segment *< u_i_, u_i_*_+1_ *>* is less than *ϵ*.
3. The number of the vertices on $\tilde{S}$ is the smallest possible.

This problem can be solved using the dynamic programming technique. We define the number of edges on $\tilde{S}(i, j)$ to be its cost. The lowest cost among all the *ϵ*-approximations for *S* is the optimal cost, denoted *c*(*i, j*). For the boundary condition that *i* = *j*, we let *c*(*i, j*) = 0. If *j > i*, there are two cases for establishing the optimal *ϵ*-approximation path.

- Case 1: $\tilde{S}(i, j)$ is the line segment (*v_i_*, *v_j_*)

This case occurs when all the distances between vertices *v_k_*, *i* ≤ *k* ≤ *j*, to
(*v_i_*, *v_j_*) are less than *ϵ*. (*v_i_*, *v_j_*) *ϵ*-approximates *< v_i_, v_i+_*_1_*, . . . , v_j_ >*, and thus
*c*(*i*, *j*) = 1

- Case 2: $\tilde{S}(i, j)$ consists of two or more line segments.

I In this case, $\tilde{S}(i, j)$ can be divided into two sub-paths, $\tilde{S}(i, k)$ and $\tilde{S}(k, j)$, where *v_k_* is a vertex on *< v_i_, . . . , v_j_ >*. Note that both $\tilde{S}(i, k)$ and $\tilde{S}(k, j)$ *ϵ*-approximate polygonal paths *< v_i_, . . . , v_k_ >* and *< v_k_, . . . , v_j_ >*. The cost of optimal *ϵ*-approximation *c*(*i, j*) is $\min_{i<k<j} (c\left( i,k \right)+c\left( k,j \right))$.

Based on the above discussion, the optimal cost can be written in the recurrence

$$c\left( i, j \right)=\left\{ \begin{aligned} 0 \\ 1 \\ \min_{i<k<j} \left( c\left( i,k \right)+c\left( k,j \right) \right) \end{aligned} \right.\begin{matrix} \mathrm{if} i=j \\ \mathrm{if}\left( v_{i},v_{j} \right) \epsilon-approximates<v_{i},\ldots,v_{j}> (1) \\ \mathrm{otherwise} \end{matrix}$$

Solving the dynamic programming, we compute the optimal *ϵ*-approximation of a polygonal path.
